# Impact of Reminders on Children’s Cognitive Flexibility, Intrinsic Motivation, and Mood Depends on Who Provides the Reminders

**DOI:** 10.3389/fpsyg.2015.01904

**Published:** 2016-01-05

**Authors:** Li Qu, Jing Y. Ong

**Affiliations:** Division of Psychology, Nanyang Technological UniversitySingapore, Singapore

**Keywords:** cognitive flexibility, intrinsic motivation, mood, kindergarten children

## Abstract

Reminding children to think about alternatives is a strategy adults often use to promote children’s cognitive flexibility, as well as children’s engagement in and enjoyment of the task. The current study investigated whether the impacts of reminders on kindergarten children’s cognitive flexibility, intrinsic motivation, and mood are moderated by who provides the reminders. Eighty-three healthy 5-year-old kindergarten children were randomly assigned to 2 (Reminder: no reminders vs. Reminders) × 2 (Agent: Tester vs. Partner) conditions. Children’s cognitive flexibility was measured via the Block Sorting Task (Garton and Pratt, 2001; Fawcett and Garton, 2005). Children reported their motivation and mood before Block Sorting, after practicing for Block Sorting, and after the actual Block Sorting. Children’s intrinsic motivation was measured by evaluating children’s choices during a period of free play after Block Sorting. The results revealed that, depending on who provides the reminders, reminding children of alternatives can influence kindergarten children’s performance on Block Sorting, children’s intrinsic motivation, and children’s self-reported mood.

## Introduction

Reminding children to think about alternatives, for example by pointing to an alternative and asking children “how about this (different) one?”, is one of the widely used methods for promoting children’s cognitive flexibility, as well as children’s engagement in and enjoyment of a task (e.g., Vandermaas et al., 2003; Garton, 2004; McGee and Schickedanz, 2007; Larkina et al., 2008; Doebel and Zelazo, 2013). However, despite the widespread use of reminders both inside and outside of research contexts, their efficacy is not a straightforward matter, as researchers have reported both facilitation and deterioration effects on children’s performance, motivation, and mood when using reminders (see review in Kaderavek and Justice, 2002; Doebel and Zelazo, 2015). In trying to illuminate this seeming contradiction, one important factor that previous work has not fully teased apart is the role an adult takes on when reminding children, namely either that of an authority or that of a partner. To fill in this theoretical gap, the current study investigated whether the impacts of reminders on children’s cognitive flexibility, intrinsic motivation, and mood are moderated by who provides the reminders, a tester or a partner.

### Development of Cognitive Flexibility During Early Childhood

Cognitive flexibility is the ability to self-initiate shifts and switches in attention as well as among mental sets, and to respond non-rigidly in order to fulfill an environmental demand (e.g., Jacques and Zelazo, 2001; Deak et al., 2004; Chevalier and Blaye, 2009; Qu et al., 2013). Although children start to develop cognitive flexibility in their infancy (e.g., Ellis and Oakes, 2006), this ability is still developing during early childhood (e.g., Zelazo et al., 1996; Beck et al., 2011). One of the widely used measures of cognitive flexibility in early childhood is the Dimensional Change Card Sorting task (e.g., Zelazo et al., 2003), a color–shape switching task. In this task, children are asked to sort bivalent cards by one dimension (e.g., color) and then switch to the other dimension (i.e., shape in this case). During switch trials, children need to suppress the tendency to use the previous dimension (in this case, color) to match pictures and activate the response tendency of using the other dimension (in this case, shape). Numerous studies have shown that by 3 years of age, most children are able to sort cards by one dimension but fail to switch to the other dimension, whereas by 5 years of age, the majority of children are able to switch (e.g., Beck et al., 2011; Doebel and Zelazo, 2015). Nevertheless, 5-year-olds still show rigidity when being tested on more complex tasks such as the advanced DCCS (Hongwanishkul et al., 2005), the Flexible Item Selection Test (Jacques and Zelazo, 2001), and the Preschool Attentional Switching Task (Chevalier and Blaye, 2008). For instance, during the Block Sorting Task (BS; Garton and Pratt, 2001; Fawcett and Garton, 2005), children are asked to sort blocks in as many different ways as possible. For kindergarten children, 12 blocks, a combination of 3 (color) × 2 (shape) × 2 (size), are used. These blocks can be sorted in six different ways; however, kindergarten children generally can make up to four correct sorts (Garton and Pratt, 2001; Fawcett and Garton, 2005), possibly because flexibility largely relies on the dorsolateral prefrontal cortex, which does not become mature until late adolescence (e.g., Bunge and Zelazo, 2006).

### Reminders May Influence Children’s Cognitive Flexibility

According to Vygotsky (1978), children’s development of cognitive flexibility is within the zone of proximal development: compared to performing a task alone, children can perform better with the guidance and support of an adult. Indeed, it has been shown that children’s performance on cognitive flexibility tasks can vary depending on the context (e.g., Deak, 2003; Deak et al., 2004; Moriguchi et al., 2007, 2010; Qu and Zelazo, 2007; Qu, 2011). Hence, in daily life, early childcare providers often try to facilitate children’s cognitive flexibility. One of the methods often used is to remind children to think about alternatives, for example by pointing to an alternative and asking children “how about this (different) one?” This method is also commonly used by researchers in psychological laboratories. Theoretically, reminding children of alternatives may influence children’s cognitive flexibility via two routes: the bottom-up route and the top-down route (Doebel and Zelazo, 2013; Perone et al., 2015).

The bottom-up stimulus-driven approach directs children to shift their attention to an alternative such as a relatively novel stimulus. It has been proposed that the task-relevant information during the pre-switch is overly active, though it then becomes irrelevant during the post-switch (e.g., Morton and Munakata, 2002; Diamond, 2006). Hence, when children shift their attention to a relatively novel stimulus, they are able to release themselves from their “stuck” attention or attention inertia, which should then allow them to further shift their mental sets and responses (e.g., Kirkham et al., 2003). Seemingly bearing out the effectiveness of the bottom-up approach for increasing cognitive flexibility, several studies have shown that distracting children with novel stimuli can increase children’s performance on the DCCS (e.g., Zelazo et al., 2003; Brace et al., 2006; Fisher, 2011).

The top-down approach is to remind children to reflect on task demands and solutions. It has been well observed that during early childhood, children generally do not engage in reflection spontaneously; hence, researchers often use questions to prompt children to reflect on the situation that the children are facing (Kirkham et al., 2003; Muller et al., 2004; Doebel and Zelazo, 2015). However, kindergarten children’s spontaneous reflections are relatively constrained. For example, by asking children to verbalize their thinking process (i.e., “think aloud”) during task switching, Karbach and Kray (2007) found that unlike 9-year-olds who mainly talked about how to respond to the target, 5-year-olds mainly labeled the perceptual features of the target. As a result, their performance during task switching was not improved. Hence, Deak et al. (2004) reminded children to think about what rule they were using for every trial before the children made a response and found that children’s performance in the rule-switching condition of the DCCS then improved.

Despite the fact that both bottom-up and top-down approaches are theoretically plausible, it is still under debate whether reminding children of the alternatives that are the opposite (or reverse) of the appropriate solution, which represents a combination of bottom-up and top-down approaches, can facilitate children’s cognitive flexibility. On the one hand, it seems plausible that a combination approach should facilitate children’s cognitive flexibility. For instance, Doebel and Zelazo (2013) have suggested that although the opposite dimension (meaning the sorting dimension that subjects were not supposed to consider at that time) was not the exact dimension that children were supposed to use for sorting, drawing their attention to that opposite dimension may still lead children to reflect how a target can be described and represented by multiple dimensions. This was indeed the case in previous training studies, which showed that after an experimenter explained to children how the same object could be perceived, described, and represented in different ways, children’s cognitive flexibility improved (Kloo and Perner, 2003; Espinet et al., 2013).

On the other hand, it has been suggested that reminding children of alternatives may actually impair children’s cognitive flexibility. When thinking about alternatives, children have to devote their relatively limited cognitive resources such as attention and working memory to these seemingly irrelevant stimuli. Such competition may impair children’s processing of the target task (Chevalier and Blaye, 2009; Marcovitch et al., 2009). For example, while processing irrelevant stimuli, children may neglect their main task goal (Marcovitch et al., 2007).

Such theoretical hypotheses notwithstanding, there has not been a study directly examining this issue. Hence, the current study aimed to explore whether reminding children of their alternatives affected their performance on sorting and switching tasks, specifically by contrasting (a) reminding children of their alternatives with (b) not reminding children at all.

### Who Provides Reminders May Moderate the Impact of Reminders on Cognitive Flexibility

Scholars such as Deak et al. (2004) have suggested that social factors associated with reminders should be taken into consideration when exploring cognitive flexibility. One of the social factors embedded in reminders is who provides the reminders. Whether in the laboratory or in daily life, an adult can provide children reminders as a person with authority or as a partner who plays with children. It is important to tease apart these two approaches, particularly since previous work has shown that these two approaches can influence children’s performance differently (e.g., Sodian et al., 1991; Hala and Russell, 2001). For instance, Hala and Russell (2001) assigned the same experimenter three different roles: in one condition, the experimenter was the instructor for the task; in another condition, she was the operator who performed the action for the child; and in yet another condition, she was the ally of the child. They randomly assigned 3-year-olds to these three conditions. It was found that children in the experimenter-as-the-ally condition outperformed their counterparts in the other two conditions on a task measuring children’s ability to inhibit their impulses. Likewise, Qu (2011) found that 3- to 5-year-old children showed better inhibitory control when a partner asked them about their solution compared to when a tester asked them about their solution. These findings indicate that a child may respond to an adult’s action differently depending on how the adult approaches the child.

### Who Provides Reminders May Moderate the Impact of Reminders on Affective Functions

Additionally, depending on who provides reminders, reminders can have different impacts on children’s affective functions such as motivation and mood.

#### Motivation

Motivation is a mental structure that drives individuals to maintain certain behaviors or conduct certain tasks (Pessoa, 2009). According to Self-Determination Theory (SDT; Deci and Ryan, 2000; Ryan and Deci, 2015), although individuals may perform a task due to external instrumental reasons such as evaluation, reward, and punishment, intrinsically individuals will perform a task so as to fulfill their innate needs for competence, autonomy, and relatedness. It seems that the same reminders may influence children’s motivation differently depending on who provides the reminders, especially when reminders are presented in the form of questions. When a tester or an adult with authority provides reminders, the reminders may signal an external evaluation, which may increase children’s extrinsic motivation but impair children’s intrinsic motivation. Contrariwise, if a partner or a teammate provides this type of reminder, then children may feel that the partner is interested in the task and is consulting them for solutions; hence, children’s intrinsic motivation increases. Generally, compared to playing alone, children who had a partner join them were more willing to play the same game again and were more able to perform the same task for a longer period of time (e.g., Simmel et al., 1969; Perlmutter et al., 1989).

#### Mood

Mood is a relatively lasting emotional state (Ashby et al., 1999; Tan and Qu, 2015). Children may feel differently about reminders depending on who tries to remind them. It has been shown that how children feel about an event depends on the association between the event and the context (e.g., Fabes et al., 1988). Children generally feel anxious if an authority gives them instructions, asks them questions, or reminds them of unfinished work (e.g., Stipek et al., 1995; Arterberry et al., 2007). By contrast, children tend to feel pleasant if a partner who shares common interests with them asks questions or makes some comment about their progress or their toys (e.g., Perlmutter et al., 1989; Garton and Pratt, 2001; Fawcett and Garton, 2005). Hence, depending on who provides the reminders, reminders can influence children’s mood differently.

### Current Study

The current study aims to investigate the impacts of an adult’s reminders on kindergarten children’s cognitive flexibility, intrinsic motivation, and mood.

To avoid any floor effect, we studied 5-year-olds. To measure cognitive flexibility over time, we used the Block Sorting Task. To measure children’s mood, we asked children to report how pleasant they felt during the pre-test, post-practice, and post-test periods. To measure children’s intrinsic motivation over time, we asked children to report how much they wanted to play with the blocks in the Block Sorting Task during the pre-test (i.e., baseline), post-practice (i.e., pre-target test), and post-test periods. Additionally, to measure children’s intrinsic motivation, after the task was finished, children’s choices during free play were recorded (e.g., Lepper et al., 1973; Deci et al., 1991). We also included a vocabulary task as a control task.

We hypothesized that the source of the reminder, being either a tester or a partner, would moderate the impact of an adult’s reminders on children’s cognitive flexibility, intrinsic motivation, and mood.

## Materials and Methods

### Participants

Eighty-three 5-year-old healthy Singaporean kindergarten children (*M* = 5.32, *SD* = 0.32, Range: 4;6–5;11; 42 girls) participated in the study. Additionally, three children participated in the study but did not sort blocks successfully during the first trial of Block Sorting; hence, their data were excluded from the final data analysis. In accordance with the Declaration of Helsinki, in all cases, parents were provided with a written description of the experiment. All parents gave written informed consent allowing their children to participate. Among them, 81.25% of parents returned the demographic information sheet, indicating that these children were from middle class families (Mothers’ education: *M* = 14.67 years, *SD* = 3.85; Fathers’ education: *M* = 14.08 years, *SD* = 4.62; Household per month income in Singapore dollars: *M* = 4860.00, *SD* = 1391.48).

### Design

In terms of cognitive flexibility, as 5-year-old children would be able to sort blocks successfully during the first trial, the design was a 3 (Test trials: second, third, vs. fourth) × 2 (Agent: Tester vs. Partner) × 2 (Reminder: Reminders vs. No reminders) within-subject and between-subject mixed design. In terms of motivation and mood, the design was a 3 (Test Time: pre-test, post-practice, vs. post-test) × 2 (Agent: tester vs. partner) × 2 (Reminder: reminder vs. no reminder) within-subject and between-subject mixed design. The conditions of Agent and Reminder were counterbalanced between the participants fully and children were randomly assigned to the conditions.

The design, test materials, and procedure were approved by the Ethics Committee, Division of Psychology, Nanyang Technological University, Singapore.

### Test Materials

#### Affective Scales of Motivation and Mood

These scales (Qu et al., 2013) included two parts. For motivation, the tester showed children a sheet of five pictures illustrating how much a cartoon character desires an item: When the character opens two arms wide at almost a 180 degree angle, it means “I really want it”; when the character opens two arms nearly 90 degrees, it means “I really want it”; when the character opens thumb and index fingers 60 degrees, it means “I want it a little bit”; when the character opens thumb and index fingers with a 1 cm gap, it means “I want it a tiny bit”; and when the character has two arms completely folded together, it means “I do not want it at all.” Children were asked to point to the cartoon picture that showed how much they wanted to play a game at the moment. Their response was scored with a 1–5 scale (1 = *really really do not want*, 2 = *really do not want*, 3 = *want a little bit*, 4 = *really want*, and 5 = *really really want*).

For the Mood Scale, the tester showed children a sheet of five cartoon faces that were, respectively, very happy, happy, neutral, upset, and crying. Children were asked to point to the face that most closely corresponded to how they felt at the moment. Their responses were scored with a 1–5 scale (1 = *very sad*, 2 = *sad*, 3 = *not happy or sad*, 4 = *happy*, and 5 = *very happy*).

#### Block Sorting

Adapted from Fawcett and Garton (2005), this task measures problem solving and cognitive flexibility. During the demonstration, the tester used four blocks, consisting of one large red cylinder, one small green cylinder, one large green rectangle, and one small red cube. These blocks could be sorted according to shape (cylinder vs. rectangle), color (red vs. blue), and size (large vs. small). For the real test, the tester used 12 blocks that can be sorted according to three basic dimensions, namely shape (circular, square), color (red, yellow, blue), and size (small, large). Additionally, these blocks can be sorted according to three complex dimensions, by color–shape, by shape–size, and by color–size. Children were asked to try to group the blocks in as many different ways as possible. Based on the literature and our pilots, during the first trial, most 5-year-olds were able to sort blocks successfully and children generally stopped trying after four trials. So, in total, children were given four trials. Children’s performances were scored in terms of the uniqueness of each trial (i.e., whether children sorted blocks into groups without repeating their previous sorting method) and the total number of unique sorts. Additionally, the dimensions children used, the complexity of the sorting dimensions, and the types of errors children made were coded, respectively, (although due to a failure of storage, only 71 out of a possible 82 children’s response photos were coded). Children’s errors were coded as 1 = *perseverated sorting* (i.e., using the sorting dimension that was used in the trial immediately before the current trial); 2 = *repeated sorting* (i.e., using the sorting dimension that was used in previous trial but not the trial immediately before the current trial); 3 = *mixed sorting dimensions* (i.e., not using any consistent sorting dimension). The inter-rater reliability, Kappa, ranged between 0.80 and 0.90.

#### Vocabulary Test

The PPVT– fourth edition (Dunn and Dunn, 2006) was used to measure children’s verbal ability. The tester showed children sets of four pictures. For each set, the tester would say a word and ask children to point at the picture that depicting the word appropriately. The test stopped at the point where children made 8 or more errors in a set of 12 words. Children’s total accurate responses were recorded as the final score.

### Procedure

**FIGURE 1:**
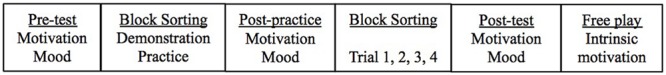
**Procedure of the current study**.

Each child was tested by one or two female experimenters in a quiet corner of the child’s kindergarten. The total testing time was about 30 min. All tests were conducted in English because English was the major language used in these kindergartens. Experimenter 1 (E1) was the main experimenter administering all the tasks. In the partner conditions, Experimenter 2 (E2) was involved as a partner for the child. Following the informed consent procedure, after chatting with the child about whether the child liked playing games and what games he or she generally played, E1 showed the child some of the toys and materials used in the current study and gave a general description of the study. Then E1 asked the child whether he or she wanted to play with her. Once the child consented to conduct the study, E1 started the following procedure (see **Figure 1**).

#### Warm-up

In the tester conditions, E1, sitting opposite the child, played with the child for 5 min; in the partner conditions, after E1 played with the child for 2 min, E2 came in and said that she wanted to play with them as well. After obtaining the child’s permission, E2 sat beside the child. The three of them played together for another 3 min.

#### Pre-test Motivation and Mood Check

E1 asked the child to illustrate how much s/he wanted to play the game by pointing to a gesture on the Motivation Scale and to illustrate how s/he felt at the moment by pointing to a facial expression on the Mood Scale. In the partner condition, after recording the child’s responses, E1 also asked E2 to indicate her motivation and mood. E2 responded in the same way that the child did.

#### Block Sorting Demonstration

In the tester conditions, E1 showed the child four blocks and told the child that these blocks could be put into different groups. E1 grouped the blocks according to one dimension. Then without naming the exact dimension she used, E1 pointed to the blocks and explained to the child, “These two blocks are the same in one way. And these two blocks are the same in another way.” She then showed the child that these blocks could be grouped in the other two manners. In the partner conditions, E1 showed both the child and E2 how to organize blocks into different groups in the same manner as in the tester conditions.

#### Block Sorting Practice

In total, there were three practice trials. The four experimental conditions varied in terms of the procedure.

##### Practice 1

In the tester conditions, E1 gave the child the blocks and asked the child to sort them into different groups: “Now, it’s your turn to play with these blocks. Please put them into groups. Let me know when you have finished. When you finish, I will take a photo of the blocks you just grouped.” In the tester-no-reminder condition, E1 did not ask the child any questions while the child was grouping the blocks. In the tester-reminder condition, 30 s after the child started to group the blocks (most children had already put two blocks in a group by now), E1 would randomly pick up one of the blocks that the child had not grouped yet and ask the child “How about this one?” and then put back the block in its original place. E1 did not make any comments or gestures for the rest of trial. In both conditions, once the child had indicated that she or he was finished, E1 took a photo of the blocks and asked the child to use a different way to group the blocks.

In the partner conditions, E1 gave the child and E2 the blocks and said “Now, it’s time for both of you to play with these blocks. Please put them into groups. Let me know when both of you have finished.” In the partner-no-reminder condition, neither E1 nor E2 asked the child any questions while the child was grouping the blocks. Once the child had indicated that they two were finished, E1 took a photo of the blocks and asked the child and E2 to use a different way to group the blocks.

In the partner-reminder condition, 30 s after the child started to group the blocks, E2, the partner, would randomly pick up a block and ask the child, “How about this one?” and then put back the block in its original place. E2 then kept quiet and made no comments afterwards.

Once the child indicated that s/he was finished, E1 took a photo of the child’s arrangement. To ensure that the child understood the instructions and procedure, E1 gave appropriate feedback and explanations according to the child’s performance. The child was asked to group the blocks again if s/he failed to group the blocks into appropriate groups. Afterward, all children grouped these blocks successfully.

##### Practice 2

Then E1 combined all the blocks and asked the child “Can you group them in another way, a different way from what you have just done?” Just as in the first practice trial, in the tester-reminder and partner-reminder conditions either E1 or E2 asked the child “How about this one?” 30 s after the child started the sorting. Then after the child had indicated that she or he was finished sorting the blocks, E1 took a photo of the child’s arrangement. When giving feedback and explanations, in addition to commenting on whether the child had successfully grouped the blocks, E1 emphasized that the second grouping method should be different from the first grouping method that the child had used. As before, the child was asked to group the blocks again if she or he failed to group the blocks successfully or to group the blocks in a new way.

##### Practice 3

The procedure was the same as in Practice 2. Eventually, all children grouped these four blocks in three different ways.

#### Post-practice Motivation and Mood Check

The procedure was the same as in the pre-test motivation and mood check.

#### Block Sorting Task

E1 put away the practice blocks and took out 12 new blocks. The procedure was the same as in the practice procedure for Block Sorting except that E1 did not provide any feedback or explanations.

#### Post-test Motivation and Mood Check

After removing the blocks, E1 measured children’s motivation and mood as she had during the pre-test motivation and mood check.

#### Post-test Intrinsic Motivation Check

E1 told the child that she needed to prepare other games. E1 took out 12 boxes of play dough of the same color as those of the blocks used in the Block Sorting. She put the play dough and the blocks in front of children, and asked the child “Which type of toy do you want to play with now, play dough or blocks (the order was counterbalanced between participants)?” E1 gave the child the chosen toys and removed the unchosen toys. Then E1 left for 1 min. In the tester conditions, the child played alone. In the partner conditions, E2 stayed and played with the child. The child scored 1 if she or he chose blocks instead of play dough.

#### Control Task

E1 returned with other test materials. E2 in the partner conditions thanked the child for playing with her and left. E1 tested the child with the PPVT.

#### End of the Study

E1 thanked the child and presented the child with a gift and a certificate as tokens of appreciation.

### Data Analysis

Separate one-way analyses of variance (ANOVAs) did not reveal any significant response differences between the children whose response photos were lost and the children whose response photos were maintained. Additionally, the general patterns of results were the same when the data of all 83 children were analyzed or when only the data of those 71 children whose response photos were maintained were analyzed. Hence, in the following section, the main results were based on the full sample (*n* = 83) except when reporting what dimensions children used, the complexity of the sorting dimension, and what errors children made, in which case the results were based on the sample with photo records (*n* = 71).

Separate independent-sample Mann–Whitney *U* Tests showed no significant performance differences in terms of gender, so the data were combined along this variable. Children’s choice during free play was not related to whether play dough or blocks were offered first. Separate ANOVAs (see **Table 1**) revealed that the four conditions were equivalent in terms of control variables such as the children’s age [*F*(3,75) = 0.05, *p* = 0.99] and vocabulary [raw scores: *F*(3,75) = 2.12, *p* = 0.11; standard scores: *F*(3,75) = 1.94, *p* = 0.13]. Pearson Chi-square tests showed that the four conditions were equivalent in terms of children’s pre-test motivation [*Z*(3,79) = 2.355, *p* = 0.50] and pre-test mood [*Z*(3,79) = 1.36, *p* = 0.72] as well as in terms of family income and parental education level. These indicated that the random assignment of children to conditions was successful. Spearman correlations (see **Table 2**) were conducted and showed that only children’s age was significantly correlated with children’s performances on the third trial of Block Sorting and children’s self-reported post-test motivation. Hence, age was included as the covariate in the further analyses.

**Table 1:** Means and standard deviations of children’s age and performance on various tasks.

		Tester-no-reminder (*n* = 21)		Partner-no-reminder (*n* = 21)		Tester-Reminder (*n* = 21)		Partner-Reminder (*n* = 20)	
		*M*	*SD*	*M*	*SD*	*M*	*SD*	*M*	*SD*
Age		64.19	3.11	64.29	3.58	62.71	4.09	64.25	4.58
Pre-test	Motivation	4.57	0.75	4.57	0.68	4.52	0.68	4.45	0.83
	Mood	4.52	0.51	4.48	0.51	4.52	0.51	4.55	0.51
Post-practice	Motivation	4.48	0.98	4.38	0.92	4.19	0.93	4.80	0.52
	Mood	4.71	0.46	4.19	0.40	4.24	0.44	4.55	0.61
Block Sorting	Second trial	0.76	0.44	0.67	0.48	0.86	0.36	0.65	0.49
	Third trial	0.67	0.48	0.52	0.51	0.52	0.51	0.70	0.47
	Fourth trial	0.57	0.51	0.14	0.36	0.24	0.44	0.50	0.51
	Total score	3.00	0.89	2.33	0.91	2.62	0.87	2.85	0.93
Post-test	Motivation	4.52	0.87	4.48	0.87	3.86	1.15	3.95	1.40
	Mood	4.76	0.44	4.38	0.74	3.95	0.87	4.35	0.75
	Free choice	0.00	0.00	0.00	0.00	0.05	0.22	0.30	0.47
PPVT		90.90	12.11	88.33	12.54	84.33	10.00	89.85	13.82

**Table 2:** Correlations (left bottom of the table) and partial correlations, after age was controlled, among children’s performance on block sorting task, motivation, and mood.

			1	2	3	4	5	6	7	8	9	10	11	12	13
1	Age														
2	Pre-test	Motivation	0.00		0.25**	0.17	0.11	-0.01	-0.02	0.04	0.00	0.35**	0.14	-0.05	0.15
3		Mood	-0.13	0.24*		-0.04	0.18	-0.15	-0.17	0.04	-0.14	0.02	0.09	-0.12	-0.05
4	Post-practice	Motivation	0.09	0.26*	-0.00		0.30**	0.23*	0.21	0.03	0.24*	0.32**	0.14	0.13	-0.21
5		Mood	-0.01	0.15	0.17	0.33**		0.23*	0.20	0.26*	0.35**	0.09	0.23*	0.26*	0.03
6	Block Sorting	Second trial	-0.05	0.01	-0.14	0.15	0.21		0.21	0.12	0.66**	0.06	0.08	-0.00	0.06
7		Third trial	0.25**	-0.01	-0.19	0.22*	0.20	0.18		0.08	0.66**	-0.05	0.31**	0.11	0.16
8		Fourth trial	0.13	0.05	0.02	0.03	0.26*	0.11	0.10		0.63**	-0.01	0.13	0.21	0.09
9		Total	0.02	0.02	-0.15	0.20	0.37**	0.60**	0.68**	0.66**		-0.01	0.27*	0.17	-0.04
10	Post-test	Motivation	0.28*	0.39**	0.03	0.39**	0.11	0.00	0.04	0.04	0.07		0.33**	-0.09	-0.02
11		Mood	0.10	0.19	0.19	0.17	0.27*	0.04	0.25*	0.12	0.21	0.35**		0.04	-0.09
12		Free choice	0.20	-0.00	-0.14	0.14	0.26*	-0.01	0.16	0.22*	0.20	0.04	-0.01		
13	Vocabulary		-0.15	0.18	-0.06	-0.25*	0.01	0.06	-0.05	0.13	0.08	-0.11	-0.07	-0.15	

*^∗^p < 0.05, ^∗∗^ p < 0.01.*

The distribution of children’s performance on each trial of the Block Sorting task was binomial. And children’s performance on the fourth trial should be different from their performance on the third trial, which should be different from their performance on the second trial, which should be different from their performance on the first trial. Due to these constraints, we chose binomial probability Generalized Linear Models (GzLMs; Dobson and Barnett, 2008) to examine how Trial, Agent, and Reminder influenced children’s performance during Block Sorting. We explored all possible models with Trial, Agent, and Reminder as the predictors, and age as the covariate. The deviance parameter estimation method was used to adjust the potential overdispersion, if the residual deviance divided by its degrees of freedom was larger than 1. The data were further examined for the expected significant interactions among Trial, Agent, and Reminder. In particular, (a) Trial effects were examined in each condition separately; (b) Agent effects were examined during each trial after splitting the data according to whether children had reminders or not; and (c) Reminder effects were examined during each trial after splitting the data according to the agent who provided the reminders. Bonferroni pairwise comparisons were used to avoid Type I, false positive, errors. Pearson Chi-squares were used to examine whether the dimensions children used for sorting and the types of errors differed by Trial, Agent, or Reminder.

Likewise, because the distributions of children’s self-reported motivation and mood were multinomial, separate multinomial ordinal logistic GzLMs were conducted with Test Time, Agent, and Reminder as the predictors, and age as the covariate. As before, the deviance parameter estimation method was used to adjust the potential overdispersion. The data were further examined for the expected significant interactions among Test Time, Agent, and Reminder. In particular, (a) Test Time effects were examined in each condition separately; (b) Agent effects were examined at each time point with Chi-square tests after splitting the data according to whether children had reminders or not; and (c) Reminder effects were examined during each trial as well as each time point after splitting the data according to the agent who provided the reminders.

Given that very few children had chosen to play blocks again, Fisher’s exact tests were conducted to examine how Agent and Reminder influenced children’s choices for free play.

## Results

### Cognitive Flexibility

#### Unique Sorting

**FIGURE 2:**
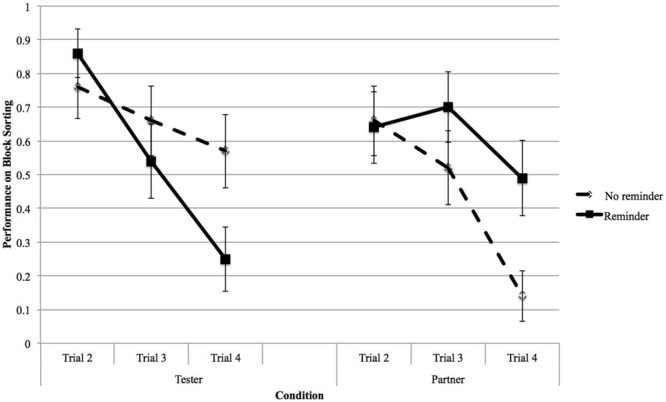
**Children’s performance on the Block Sorting Task by trial and condition**.

In terms of whether children made unique sorts after their initial sort, in the final model, there were significant interaction effects among Trial, Agent, and Reminder conditions [χ^2^(11,249) = 41.91, *p* < 0.001]; see results in **Figure 2**. The results showed that Trial effects were significant in the tester-reminder condition [χ^2^(2,63) = 17.60, *p* < 0.001], indicating that children’s performances during the second trial were significantly better than their performances during the third trial (*M*_difference_ = 0.33, *SE* = 0.13, *p* = 0.037) and the fourth trial (*M*_difference_ = 0.62, *SE* = 0.12, *p* < 0.001). Additionally, the Trial effects were significant in the partner-no-reminder condition [χ^2^(2,63) = 13.53, *p* = 0.001], showing that children’s performances during the second trial (*M*_difference_ = 0.52, *SE* = 0.13, *p* < 0.001) and the third trial (*M*_difference_ = 0.38, *SE* = 0.13, *p* = 0.013) were significantly better than their performances during the fourth trial. These findings revealed that children’s performance on Block Sorting decreased significantly in the tester-reminder and partner-no-reminder conditions.

Among the children working with the tester alone, during the third trial [χ^2^(1,60) = 3.86, *p* = 0.049] and the fourth trial [χ^2^(1,60) = 8.31, *p* = 0.004], there were significant Reminder effects, showing that those whose tester reminded them of their options performed significantly worse than those whose tester did not so remind them (third trial: *M*_difference_ = 0.24, *SE* = 0.12; fouth trial: *M*_difference_ = 0.32, *SE* = 0.15). During the fouth trial, the Reminder effect was also significant among the children working with a partner [χ^2^(1,41) = 6.27, *p* = 0.012], showing that those whose partner reminded them of their options performed significantly better than those whose partner did not remind them (*M*_difference_ = 0.36, *SE* = 0.12). These findings indicated that depending on the context, reminders influenced children’s performance on Block Sorting differently.

Furthermore, during the fouth trial, when there were no reminders, there was a significant Agent effect [χ^2^(1,58) = 12.04, *p* = 0.001], showing that children who worked with the tester alone performed significantly better than did those counterparts who had a partner sitting beside them (*M*_difference_ = 0.43, *SE* = 0.12). This pattern was not significant among the children who were given reminders [χ^2^(1,41) = 3.07, *p* = 0.08; *M*_difference_ = -0.26, *SE* = 0.15]. These findings indicated that without reminders, the presence of a partner decreased children’s performance.

#### Sorting Dimensions

The results (see **Table 3**) showed that during the second trial, there was a Reminder effect [χ^2^(6,71) = 14.18, *p* = 0.028] on what particular dimension children used for sorting, as there were more children in the reminder condition (30%) who sorted blocks by shapes than in the no-reminder condition (6%). This effect was significant among the children who interacted with the tester [χ^2^(6,38) = 12.59, *p* = 0.05], and not among the children who had a partner sitting beside them [χ^2^(6,33) = 7.79, *p* = 0.25]. These findings suggested that during the second trial, the tester’s reminder “how about this one?” may have led children to focus on the shape dimension.

**Table 3:** Sorting dimensions and types of errors during block sorting.

		Tester-no-reminder (*n* = 20)			Partner-no-reminder (*n* = 15)			Tester-reminder (*n* = 18)			Partner-reminder (*n* = 18)		
Dimension used		Second	Third	Fourth	Second	Third	Fourth	Second	Third	Fourth	Second	Third	Fourth
Wrong		0	2	1	2	0	4	1	2	4	3	1	4
Simple	Shape	1	2	3	1	3	2	7	0	2	5	3	2
	Color	4	2	3	1	2	3	3	2	1	4	1	1
	Size	5	3	4	4	3	0	4	6	5	1	4	1
Complex	Shape–Color	1	4	0	2	1	0	2	2	2	1	4	4
	Shape–Size	6	3	6	2	5	5	1	6	3	3	2	3
	Color–Size	3	4	3	3	1	1	0	0	1	1	3	3
Error Type	Perseverated	2	5	4	3	3	2	2	4	6	2	1	3
	Repeat	0	0	5	0	5	3	0	2	3	0	3	3
	Mixed	1	2	1	1	0	5	1	2	3	3	1	4

#### Complexity of Sorting Dimensions

There was a significant age effect [χ^2^(1,213) = 5.63, *p* = 0.018] as older children used more complex sorting dimensions than younger children, but there were no significant main effects of Trial, Reminder, or Agent, or any significant interaction effects.

#### Sorting Errors

Children in different conditions seemed to make different types of errors across trials. First, there was a significant Trial effect in the tester-no-reminder condition [χ^2^(6,60) = 13.62, *p* = 0.034]: children made more repeat sorting errors during the fourth trial, compared to other trials. Second, there was a significant Trial effect in the partner-no-reminder condition [χ^2^(6,45) = 14.44, *p* = 0.025]: children made more repeat sorting errors during the third and more mixed sorting errors during the fourth trial, compared to other trials. The Trial effect was not so significant in the other two conditions. Third, there was a significant Agent effect during the third trial among the children who were not reminded [χ^2^(3,35) = 8.77, *p* = 0.033]: more children in the partner-no-reminder condition made repeat errors compared to those in the tester-no-reminder condition. These findings indicated that children in the partner-no-reminder condition, in particular, tended to make repeat sorting errors.

### Self-Reported Motivation

There were no significant Test Time, Agent, Reminder, or age effects on children’s self-reported motivation.

### Self-Reported Mood

The final model showed a significant interaction effect among Test Time, Agent, and Reminder conditions [χ^2^(11,249) = 33.12, *p* = 0.001]. The Test Time effects were further examined among the four conditions. The results revealed significant Test Time effects in the tester-reminder condition [χ^2^(2,63) = 8.00, *p =* 0.018], showing that children reported feeling significantly more pleasant during the pre-test period as compared to the post-test period [*B* = 1.88, *SE* = 0.79, 95% *CI* = (0.33,3.43), χ^2^(1,42) = 5.66, *p =* 0.017]. These findings indicated that only children in the tester-reminder condition experienced decreasing feelings of pleasantness across the test period.

Additionally, among the children who interacted with the tester alone, there were significant Reminder effects during both the post-practice period [χ^2^(1,60) = 14.23, *p* < 0.001] and the post-test period [χ^2^(1,60) = 14.56, *p* < 0.001]. According to the parameter estimates, children in the tester-no-reminder condition reported feeling more pleasant compared to those in the tester-reminder condition [post-practice: *B* = 2.12, *SE* = 0.62, 95% *CI* = (0.90,3.33), χ^2^(1,60) = 11.67, *p =* 0.001; post-test: *B* = 2.01, *SE* = 0.56, 95% *CI* = (0.92,3.12), χ^2^(1,60) = 12.96, *p* < 0.001]. Among the children who had a partner sitting beside them, this pattern was reversed during the post-practice period [χ^2^(1,41) = 5.89, *p* = 0.015], as children in the partner-no-reminder condition reported feeling significantly less pleasant compared to those in the partner-reminder condition [*B* = -1.60, *SE* = 0.75, 95% *CI* = (-3.06,-0.14), χ^2^(1,41) = 4.60, *p =* 0.032]. This pattern was not significant during the post-test period [χ^2^(1,41) = 0.49, *p* = 0.83]. These findings indicated that depending on the context, reminders influenced children’s feeling of pleasantness differently.

Among the children who were not reminded, there were significant Agent effects during both the post-practice period [χ^2^(1,58) = 11.72, *p* = 0.001] and the post-test period [χ^2^(1,58) = 3.97, *p* = 0.046]. According to the parameter estimates, children who worked with the tester alone reported feeling more pleasant compared to those who had a partner sitting beside them [post-practice: *B* = 1.94, *SE* = 0.61, 95% *CI* = (0.74,3.14), χ^2^(1,58) = 10.01, *p =* 0.002; post-test: *B* = 1.06, *SE* = 0.552, 95% *CI* = (-0.01,2.13), χ^2^(1,58) = 3.75, *p* = 0.053]. However, among the children who were reminded, during the post-practice period this pattern was reversed [χ^2^(1,41) = 4.90, *p* = 0.027], as children who worked alone with the tester reported feeling significantly less pleasant compared to those who had a partner joining them [*B* = -1.46, *SE* = 0.70, 95% *CI* = (-2.83,-0.09), χ^2^(1,41) = 4.36, *p =* 0.037]. This pattern was not significant during the post-test [χ^2^(1,41) = 2.27, *p* = 0.13]. These findings indicated that without reminders, children felt more pleasant working alone than teaming up with a partner; however, with reminders, children felt more pleasant teaming up with a partner than working alone.

### Intrinsic Motivation

There were significantly more children in the partner-reminder condition (*n* = 6) who chose to play with the blocks during free play as compared to the tester-no-reminder (*n* = 0), the tester-reminder (*n* = 1), and the partner-no-reminder (*n* = 0) conditions. Fisher’s exact tests showed that among the children who had a partner sitting beside them while performing Block Sorting, there was a significant Reminder effect (*p =* 0.009): more children whose partner reminded them of their options chose to play blocks again, as compared to those children whose partner did not remind them of their options. Additionally, among the children who were reminded of their options, there was an Agent effect (*p =* 0.045), in that more children whose partner reminded them of their options chose to play blocks again, as compared to those children whose tester reminded them of their options.

### Correlations Among Children’s Cognitive Flexibility, Motivation, and Mood

The results (see **Table 2**) showed that after controlling for children’s age, children’s Block Sorting total score significantly correlated with children’s post-practice self-reported motivation, post-practice self-reported mood, and post-test self-reported mood, suggesting that potentially cognitive flexibility, motivation, and mood may interact with each other.

## Discussion

**FIGURE 3:**
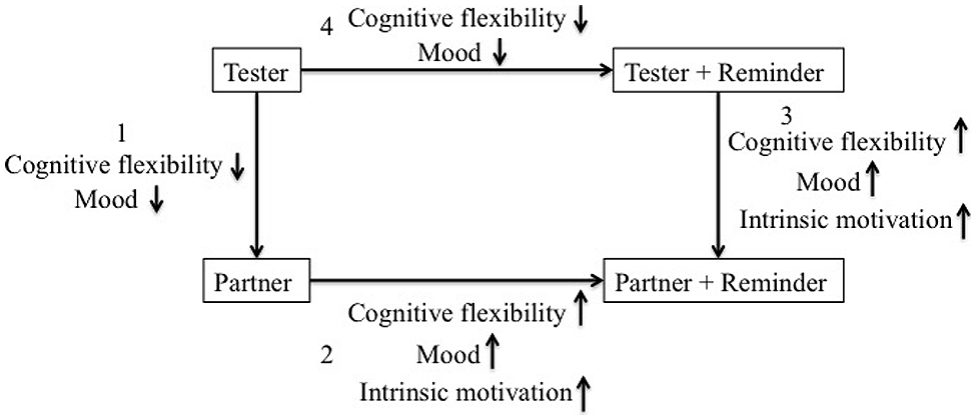
**Summary of findings on between-condition differences.** (1) Without reminders, children performed better on Block Sorting and reported feeling more pleasant in the tester-no-reminder condition than in the partner-no-reminder condition. (2) Among the children who had a partner sitting beside them when they interacted with the tester, children performed better on Block Sorting, reported feeling more pleasant, and appeared to be more intrinsically interested in blocks in the partner-reminder condition than in the partner-no-reminder condition. (3) With reminders, children performed better on Block Sorting, reported feeling more pleasant, and appeared to be more intrinsically interested in blocks in the partner-reminder condition than in the tester-reminder condition. (4) Among the children who interacted with the tester alone, children performed better on Block Sorting and reported feeling more pleasant in the tester-no-reminder condition than in the tester-reminder condition.

The results (see **Figure 3**) showed that to a certain degree, depending on who provided the reminders, reminders did influence children’s cognitive flexibility, mood, and intrinsic motivation. In particular, we found (a) a significant interaction effect of Trial, Reminder, and Agent on children’s cognitive flexibility, (b) a significant interaction effect of Test Time, Reminder, and Agent on children’s mood, and (c) a significant condition difference in terms of children’s intrinsic motivation as measured via children’s choice during free play. These results highlight the importance of considering the social context when examining the effects of reminders on children’s performance.

### Reminders Influenced Children’s Cognitive Flexibility

Supporting our hypothesis, the results revealed that depending on who prompted children, reminding children of their alternatives influenced children’s performance on Block Sorting. On the one hand, we saw a detrimental effect of reminders for children who conducted Block Sorting with a tester. Specifically, the results showed a significant between-subject difference: children in the tester-no-reminder condition outperformed those in the tester-reminder condition during the third and fourth trial of Block Sorting. Furthermore, the results showed a significant within-subject change among children in the tester-reminder condition: children’s performance on Block Sorting decreased significantly from Trial 2 to Trial 3 and Trial 4. These findings indicate that reminders provided by the tester impaired children’s cognitive flexibility during the final stage.

Additionally, it appears that during the second trial, the reminder provided by the tester made children use the shape dimension more often during sorting compared to other dimensions. It seems that the “one” used in the question “how about this one” may lead children to pay attention to the shape dimension, a whole-object constraint, as proposed by Markman (1990). Furthermore, unlike those children in the tester-no-reminder condition who made more repeat errors during the fourth trial, children in the tester-reminder condition made various types of errors during all trials. It is possible that the reminders posed by the tester prompted children to think more about the opposite blocks that the tester was indicating. Such a process might compete with the cognitive resources needed for children to engage in set shifting, thus impairing children’s cognitive flexibility. This proposal is in line with the findings of Chevalier and Blaye (2009) as well as those of Marcovitch et al. (2009).

On the other hand, the results revealed that reminders had a buffer effect among children who conducted Block Sorting in the presence of a partner. Particularly, there was a significant between-subject difference: children in the partner-reminder condition outperformed their counterparts in the partner-no-reminder condition during the fourth trial of Block Sorting, a finding which is consistent with Deak et al. (2004) report. Moreover, the results showed a significant within-subject change among children in the partner-no-reminder condition: these children decreased their performance significantly from Trial 2 and 3 to Trial 4. Further error analyses revealed that in this partner-no-reminder condition, children tended to make more repeat sorting and mixed sorting errors. It appears that, as would be consistent with the literature, the presence of a partner who simply sat beside a child may have decreased children’s performance – a social loafing effect (Latane et al., 1979). However, these patterns did not appear for children in the partner-reminder condition. These findings suggest that reminders provided by the partner may help children stay on track with their target task, focus on their main overall goal, or improve their working memory momentarily.

### Reminders Influenced Children’s Mood

Partially consistent with our hypotheses, the results showed that depending on who provided the reminders, reminders either made children feel more pleasant or less pleasant. Reminders had a detrimental effect on the children who interacted with the tester alone. In particular, there were significant between-subject differences: During both the post-practice and post-test periods, significantly fewer children whose tester gave them reminders reported feeling pleasant, as compared to their counterparts whose tester did not give them reminders. Additionally, significant within-subject changes only appeared in children whose tester gave them reminders: These children reported feeling less pleasant during the post-test period than during the pre-test period. These results are similar to previous findings stating that when interacting with an authority, especially under the evaluation of that authority, kindergarten children reported unpleasant feelings (e.g., Stipek et al., 1995).

By contrast, the results showed a significant facilitation effect for reminders among children who had a partner sitting beside them. Specifically, there was a significant between-subject difference during the post-practice period: more children who received reminders from their partner reported feeling pleasant, as compared to their counterparts who did not receive such reminders from their partner. These findings are in line with past results stating that the presence of an active partner made children enjoy a task more (e.g., Fawcett and Garton, 2005).

However, contrary to our expectations, during the post-test period there was no significant difference between the partner-reminder and partner-no-reminder conditions as to the children’s self-reported feelings of pleasantness. Additionally, there was no significant within-subject increase in terms of children’s feeling of pleasantness among children whose partners reminded them of alternatives. These findings suggest that reminders only increase children’s mood to a relatively limited degree. It is possible that the partners in the current study were not as active as those partners in previous studies, who not only reminded children but also asked questions, commented on children’s performance, or made specific suggestions (e.g., Fawcett and Garton, 2005).

### Reminders Influenced Children’s Intrinsic Motivation

Partially consistent with our hypotheses, the results showed that among the children who had a partner sitting beside them, there were significantly more children in the partner-reminder condition who chose to play blocks again during their free play, as compared to those in the partner-no-reminder condition. It is possible that, as stated in the SDT (e.g., Ryan and Deci, 2015), the partner’s reminders satisfied children’s needs for competence, autonomy, and relatedness, and therefore, children’s intrinsic motivation was increased. These results are consistent with the literature which asserts that when children had a partner playing with them, asking them questions, and commenting on their behaviors, they were able to continue a task for a longer period of time than when they were working on a task alone (e.g., Fawcett and Garton, 2005).

However, contrary to our expectations, reminders did not influence children’s self-reported motivation. In fact, the results suggested that children’s self-reported motivation did not change over time. Although we asked children to report how they felt about playing, it is possible that children at this age were not able to differentiate their intrinsic motivation from their extrinsic motivation. Hence, children’s choice during free play may be a more sensitive measure of children’s intrinsic motivation (Deci et al., 1991).

### Social Loafing and Social Facilitation Effects

We found that among the children who did not receive reminders, there were fewer children in the partner-no-reminder condition who successfully sorted blocks during the fourth trial of the Block Sorting Task and fewer children who reported feeling very pleasant, as compared to their counterparts in the tester-no-reminder condition. By contrast, among the children who did receive reminders, children in the partner-reminder condition performed better during the fourth trial of the Block Sorting Task than did their counterparts in the tester-reminder condition, which is consistent with previous findings that children performed better when the experimenter took the role of partner than when the experimenter took the role of instructor (Hala and Russell, 2001; Qu, 2011). Furthermore, compared to their counterparts in the tester-reminder condition, children in the partner-reminder condition reported feeling more pleasant, and appeared to be more interested in playing blocks during free play. The former finding may be related to the social loafing effect (Latane et al., 1979), and the latter finding may be related to the social facilitation effect, which states that the presence of other people can increase individuals’ performance (Zajonc, 1965).

According to Harkin’s (1987) evaluation theory, in cases where individuals believe that their performance is not under evaluation, they tend to perform worse when teaming up with a partner than they do when conducting the task individually. Social loafing can be decreased or even reversed to become social facilitation if individuals believe that their performance is under evaluation and that good performance will be rewarded afterward. Arterberry et al. (2007) found this exact dynamic in their work with 5-year-olds. Although we did not explicitly inform children that their performance would be evaluated, children may have interpreted the situation as one of evaluation depending on who provided the reminders to them. Future studies can examine this issue further.

## Summary

Integrating the above findings, we can conclude that reminding children of alternatives can influence not only children’s cognitive flexibility but also children’s mood and intrinsic motivation. Among children who interacted with a tester, the tester’s reminders impaired children’s cognitive flexibility and mood. Among children who had a partner sitting beside them while interacting with a tester, a partner’s reminders served mainly as a buffer to protect children from decreasing their cognitive flexibility, mood, and intrinsic motivation while they had a partner sitting beside them and doing nothing. Additionally, the results also showed that children’s cognitive flexibility, motivation, and mood correlated with each other significantly.

These results suggest several possible routes by which reminders of alternatives could have influenced children’s cognitive flexibility. The first possible influence could come through a cognitive route. Although in our study both the tester and the partner gave the same type of reminders, the consequences were different. When the tester pointed to seemingly irrelevant blocks, children may stop what they were thinking about and shift their attention to these blocks. However, these blocks did not bring any direct insights into the solution. Thus, these reminders did not improve children’s performance; rather they may make children temporarily neglect their main task goal and impair their working memory (e.g., Marcovitch et al., 2007; Chevalier and Blaye, 2008). On the other hand, when the partner pointed to seemingly irrelevant blocks, children may not take the suggestions seriously, and thus they may not allocate too much of their cognitive resources to these irrelevant blocks. Hence, their performance was not impaired.

The second possible influence on cognitive flexibility could come through an affective route. Compared to children whose tester did not provide any reminders, children whose tester provided some reminders may make children feel anxious, which may impair children’s performance especially when they were performing a relatively complex task (Arterberry et al., 2007). Compared to children whose partner simply sat beside them, children whose partner provided some reminders of alternatives may make children feel more pleasant. This kind of mildly positive mood may facilitate children’s cognitive flexibility. It is possible that positive mood may also improve children’s other cognitive functions such as perspective taking, goal setting, working memory, remote association, and attention shifting, which may further facilitate children’s cognitive flexibility (e.g., Ashby et al., 1999).

The third possible influence on cognitive flexibility could come through an intrinsic motivation route. It is possible that reminders of alternatives provided by a partner may increase children’s intrinsic motivation, which may increase children’s cognitive flexibility as well as children’s feeling of pleasantness. By contrast, reminders of alternatives provided by a tester may impair children’s cognitive flexibility as well as children’s feeling of pleasantness. This proposal is in line with the SDT (Ryan and Deci, 2015) and previous findings (Qu et al., 2013).

Last, it is also possible that reminders of alternatives influenced children’s cognitive flexibility via all three routes. Therefore, future studies with other cognitive, motivational, and affective measures are needed to further examine these possibilities.

## Implications, Limitations, and Future Directions

Although reminding children of alternatives is often used in laboratories by psychologists as well as in teaching settings by parents and early care providers, to our knowledge, the current study is the first experiment systematically and comprehensively investigating the impacts of this type of reminder on children’s cognitive flexibility, intrinsic motivation, and mood. In addition to bringing insights into the theoretical debate as to whether reminding children of alternatives influences children’s cognitive flexibility, motivation, and mood (e.g., Vandermaas et al., 2003; Garton, 2004; McGee and Schickedanz, 2007; Larkina et al., 2008; Doebel and Zelazo, 2013), our findings also indicate that social context can likewise influence children’s cognitive flexibility, intrinsic motivation, and mood. These results that children can perform will under some situations but not so well under other situations are consistent with previous findings that kindergarten children are sensitive to task demands and that they begin to make context-appropriate responses, which is cognitive flexibility in a broad sense (e.g., Deak, 2003; Deak et al., 2004; Moriguchi et al., 2007; Qu, 2011). Methodologically, our results emphasize the importance of examining children’s motivation and mood states while investigating children’s cognitive functions and behaviors. Additionally, we found that both reminders by a tester and the presence of a partner who did not make any contribution can impair children’s cognitive flexibility and mood, suggesting that such practices should be avoided in daily life.

Nevertheless, our study also has limitations. For example, we assumed that the question “how about this one?” could lead children to shift their attention and engage in self-reflection, but we did not measure such attention shifts and self-reflections directly. Future studies can include tasks measuring children’s attention and reflections to verify our assumption. Furthermore, although most children did not immediately use the blocks to which the tester or partner pointed, we did not record whether or not they used these blocks during the particular trials eventually. We also did not interview children concerning how they thought about the reminders. Such information might help to clarify the exact mechanisms by which reminders influence children’s performance. Additionally, due to methodological constraints, we could not record children’s motivation and mood simultaneously during their Block Sorting Task. Future studies can record children’s physical and electrophysiological responses to further examine the dynamic interactions between children’s cognitive and affective functions. In addition, future studies can examine whether our findings are generalizable to other age groups such as 3-year-olds. Moreover, future studies can examine whether other types of reminders and other types of relationships between children and adults can have similar impacts on children.

Lastly, our results indicate that reminders, depending on who poses them, can influence children’s cognitive flexibility, intrinsic motivation, and mood momentarily. It is unclear whether these momentary changes can transfer to other contexts. It seems plausible. For example, Qu and Lim (in press) have found that an adult can not only influence children’s cognitive and affective responses in one situation, but can also influence children’s approach to a subsequent new situation, even in the absence of the particular adult. Additionally, previous training studies have shown providing several sessions of explanations, feedback, and reminders can not only improve children’s cognitive flexibility but also children’s understanding of mental states (e.g., Espinet et al., 2013). Nevertheless, future studies are still needed to further examine whether there are any carry-over and longitudinal effects associated with reminders.

## Conclusion

The current study has revealed that, depending on who provided reminders, reminding children of alternatives can influence kindergarten children’s performance on Block Sorting, children’s self-reported mood, and children’s intrinsic motivation. In particular, the results showed that among the children who were tested by the tester, children whose tester provided reminders performed worse on Block Sorting and reported feeling less pleasant during the post-practice and the post-test periods, compared to children whose tester did not remind provide reminders. By contrast, among the children who were tested by the tester in the presence of a partner, children whose partner provided reminders performed better on Block Sorting, reported feeling more pleasant during the post-practice period, and were intrinsically more interested in playing blocks, compared to children whose partner did not provide reminders. Theoretically, the findings shed some light on the debate as to whether reminding children of alternatives facilitates or impairs children’s cognitive flexibility, and also illustrate that, depending on their source, reminders can influence children’s cognitive flexibility, intrinsic motivation, and mood. Practically, our results provide some guidance on how to improve children’s cognitive flexibility in the psychological laboratory and in daily life.

## Author Contributions

LQ initiated and designed the study, supervised the data collection, analysed the data, and wrote the manuscript. JO collected the data, conducted preliminary data analysis, and drafted the manuscript.

## Conflict of Interest Statement

The authors declare that the research was conducted in the absence of any commercial or financial relationships that could be construed as a potential conflict of interest.
